# Monitoring Behaviour in African Elephants during Introduction into a New Group: Differences between Related and Unrelated Animals

**DOI:** 10.3390/ani11102990

**Published:** 2021-10-18

**Authors:** Franziska Hörner, Ann-Kathrin Oerke, Dennis W. H. Müller, Uta Westerhüs, Idu Azogu-Sepe, Jiri Hruby, Gela Preisfeld

**Affiliations:** 1 Fakultät für Mathematik und Naturwissenschaften, Zoologie und Didaktik der Biologie, University of Wuppertal, Gaußstraße 20, D-42119 Wuppertal, Germany; apreis@uni-wuppertal.de; 2 Endocrinology Laboratory, German Primate Centre, Kellnerweg 4, D-37077 Goettingen, Germany; akoerke@dpz.eu; 3 Zoological Garden Halle (Saale), Fasanstr. 5a, D-06114 Halle (Saale), Germany; dennis.mueller@zoo-halle.de; 4 Opel-Zoo Kronberg, Am Opel-Zoo 3, D-61476 Kronberg im Taunus, Germany; uta.westerhues@opel-zoo.de; 5 Serengeti-Park Hodenhagen, Am Safaripark 1, D-29693 Hodenhagen, Germany; i.azogu@serengeti-park.de; 6 ZOO Dvůr Králové, Štefánikova 1029, C-544 01 Dvůr Králové nad Labem, Czech Republic; j.hruby@zoodvurkralove.cz

**Keywords:** African elephant, zoo elephants, unification, reunification, communication, behaviour, *Greeting Ceremony*

## Abstract

**Simple Summary:**

African elephants are highly social animals that perform a so-called *Greeting Ceremony* in the wild when meeting elephants they are familiar with but have not seen for a certain timespan. Until now, it has not been known whether zoo elephants also show this unique behaviour. Therefore, this study was designed around the reunifications of two mother–daughter pairs that had been separated for 2 and 12 years, and two unifications of unrelated elephants, as a comparison. First contact was conducted in a protected setting, i.e., there was a fence between the animals to prevent possible fighting. Signs of the *Greeting Ceremony* shown by the elephants, the distance they kept to the separating fence, and the time until the elephants’ trunks touched for the first time were observed. The results demonstrate that the related elephants showed all behavioural characteristic of the *Greeting Ceremony*, kept close to the fence, and touched trunks after only a few seconds, while elephants that were not familiar with each other did not show a full *Greeting Ceremony*, stayed further from the fence, and touched trunks for the first time only after several minutes upon meeting. This study testifies that zoo elephants show the same typical social behaviour known from wild elephants (namely the *Greeting Ceremony*) and, therefore, behave species-specific. It also confirms the strong family bonds of elephants and the cognitive abilities of elephants, specifically their long-term social memory.

**Abstract:**

The introduction of elephants into new groups is necessary for breeding programmes. However, behavioural studies on the reactions of these animals at first encounters are missing. In the present study, female African elephants *(Loxodonta africana)* living in zoos were observed during unifications with unfamiliar elephants (introduction of two to one females and one to two females; *n* = 6) and reunifications with related elephants (two mother–daughter-pairs; *n* = 4) that were separated for 2 and 12 years, respectively. First encounters of the elephants were observed and recorded by scan sampling. The parameters measured were (a) signs of the characteristic *Greeting Ceremony*, (b) distance to the fence separating the elephants during first contact, and (c) time until trunks touched for the first time. The data were statistically analysed with SPSS. The results showed that related elephants performed a full *Greeting Ceremony* on reunifications. Unrelated elephants only expressed a minor greeting. During first encounters, related elephants predominantly showed affiliative behaviour (*p* = 0.001), whilst unrelated elephants expressed more agonistic behaviour (*p* = 0.001). The distance to the fence was significantly smaller for related elephants than for unrelated elephants (*p* = 0.038). first contact of trunks occurred on average after 3.00 s. in related elephants and 1026.25 s. in unrelated elephants. These findings indicate that related elephants recognise their kin after up to 12 years of separation, meet them with a full *Greeting Ceremony* during reunification, and seek contact to the related elephant, while unrelated elephants are hesitant during unifications with unfamiliar elephants and express more agonistic behaviour. The results testify that zoo elephants show the same species-specific social behaviour as their conspecifics in the wild. It also confirms the cognitive abilities of elephants and the significance of matrilines for breeding programmes.

## 1. Introduction

### 1.1. Elephant Communication

#### 1.1.1. Greeting Ceremony

Known to be highly sensitive mammals with a complex social structure and extraordinarily developed ways of communication, elephants and their behaviour have been a frequent topic of research [1,2,3,4,5,6,7]. However, it is mainly olfactory [8,9,10,11,12] and auditory [4,13,14,15,16,17,18,19] communication that has been investigated [7]. While sexual and breeding behaviour and communication are well-represented [20,21,22,23,24,25,26], the so-called *Greeting Ceremony* [7] with its enormous olfactory, visual, tactile, and acoustic aspects is investigated poorly for ex situ living African elephants, so far.

While elephants usually greet other elephants by flapping their ears, lifting the head, and sometimes touching the head of the other individual with their trunk (referred to as *Little Greeting*) [27], the *Greeting Ceremony* is much more complex and usually restricted to interactions between closely related elephants [7]. The ethogram in Table 1 shows the behavioural items that form the *Greeting Ceremony* [7,18,28,29,30,31].

#### 1.1.2. Affiliative and Agonistic Communication

Communication expressed by behaviours during greetings can be further classified as affiliative, agonistic, and neutral [7,18,28,29,30,31]. The neutral behavioural *eating/drinking* is listed under (re)unification, as it is used as an indicator for stress in the animals. Since stress induces a rise in cortisol, it operates anorexiant [32,33,34,35,36,37]. Thus, only animals that are more relaxed during (re)unification are expected to show this behaviour. Table 2 lists all behaviours included in this study.

### 1.2. Elephant Transfers 

#### 1.2.1. Unifications

The management of the African elephant population in European zoos has to maintain a defined birth rate to ensure the viability of the population and its biodiversity [38,39,40,41,42,43,44,45]. Thus, elephant transfers to bring animals in potential breeding situations are common. This applies mostly for males, but when space becomes limited, sometimes females need to be transferred as well [44,45]. Hence, elephants have to be acquainted with new housing conditions; new surroundings; and most importantly, new herd members. Those unifications of unrelated elephants are very difficult situations when handling elephants [38,44]. Maintaining such a situation with the right caution is essential for the successful joining of different elephant groups. Knowing how elephants behave on such occasions is highly beneficial to prevent possible aggressive behaviour or a failure in merging the two groups.

#### 1.2.2. Reunifications

Nowadays, European zoos seek to keep elephants in herd structures similar to the way elephants live in the wild [38,40,45], with cows living with their female offspring in multigenerational herds [37,38]. In the past, however, occasional separation of mothers and daughters took place in European zoos [42]. Given the information from the wild, a reunification of related individuals might provide different results in comparison with unification of unrelated animals, with possibly different behaviour in the elephants involved. Scientific understanding of the underlying factors during (re)unification are important for the preservation of the species-specific social structure and the well-being of African elephants in modern zoos.

### 1.3. Aims of the Study

The so-called *Greeting Ceremony* is an indicator for elephants’ recognition of and a friendly attitude towards each other [7,31]. Whilst frequently described for wild-ranging elephants [28,29], to the best of our knowledge, there is no empirical data on the *Greeting Ceremony* for zoo-living elephants. This study aims to investigate the behaviour of related and unrelated African elephants at first encounters during (re)unification and the possible expressing of the characteristic *Greeting Ceremony* in a zoo environment. Confirming that zoo-socialised elephants express the same social behaviour and make use of the same ways of communication as in situ living individuals is of particular importance, as the zoos and studbooks aim to ensure a species-specific development of the zoo-bred African elephants [38,39,40,41,42,43,44,45].

It can be expected that elephants that were separated for a certain timespan will make use of the *Greeting Ceremony* on reunification, while unfamiliar elephants will not show signs of a *Greeting Ceremony* when unified [7]. Hypothesising that related elephants will easily be adjoined and show intense emotional behaviour on reunification, it would give evidence of the long-term memory of this species. Recognition of a related animal after a longer period of separation, using the *Greeting Ceremony*, would attest to this particular ability in African elephants.

## 2. Material and Methods

### 2.1. Animals

In the framework of the European Endangered Breeding Programme (EEP) for the African elephant, recommendations were made to transfer a daughter (Panya) to her mother (Bibi) and a mother (Pori) back to her daughter (Tana). It was also recommended to transport two unrelated cows (Lilak and Kariba) to another place with another single elephant (Zimba) and one unrelated elephant (Drumbo) to two unrelated cows (Saly and Umbu).

Even though most of the elephants were born in the wild, they were transferred to European zoos at a young age and socialised under zoo conditions. 

For more detail on the elephants, see Table 3.

All unifications and reunifications took place under the same (testing) conditions. The sample size for related elephants was *n* = 4, and the sample size for unrelated elephants was *n* = 6. The sample size for reunifications of the related and unrelated elephant groups waws *n* = 2.

During the unification of Zimba with Lilak and Kariba, Zimba was in her stable when Lilak and Kariba were released separately into the enclosure next to hers; therefore, two data sets are presented (Zimba&Lilak and Zimba&Kariba). During the unification of Drumbo with Umbu and Saly, Drumbo was in the stable and Umbu and Saly entered the enclosure next to hers together, resulting in one data set. During the reunifications, the daughters (Tana and Panya) were in their enclosures and their mothers (Pori and Bibi) entered the adjacent enclosure.

### 2.2. Ethological Data Collection

All behaviours of the elephants on first protected meeting through a fence were documented utilising the ethogram (Table 1) according to scan sampling by the same human observer [46,47,48,49,50,51], focusing on signs of behaviour characteristic for the *Greeting Ceremony* [7,18,28,29,30,31,52,53]. Acoustic signals (trumpets, rumbles, and roars) were noted and specified when heard. Additionally, the ethogram differentiated between signs of agitation related to excitement (affiliative connotation) and signs of agitation related to fear (agonistic connotation) [7,27,45,46]. Procedures were observed while elephants were still separated through a fence, though in tactile, visual, auditory, and olfactory contact, as first meetings during the introduction of new herd members were performed with a barrier for safety reasons. Even though observation times ranged between 35 to 78 min, most behaviours occurred in the first 30 min. Therefore, only the first 30 min were used for analyses.

The distance that the elephants kept to the fence throughout the (re)unification was measured in meters to assess their willingness to touch the other individual [47]. The distance was based on direct contact (meaning tactile contact to the fence or animal) or distance of <1 m, 1–2 m, 3–4 m, and >4 m. The elephants’ distance to the fence was recorded every 10 s during the (re)unification.

For all elephants, the first moment of tactile contact during (re)unification was determined and is referred to as *first contact of trunks* throughout this paper. This indicator was used to describe the willingness of the elephants to reach for and touch the other elephant and for their curiosity [47].

The sets of data for behaviour and distance to the fence were classified numerically [54,55]. Statistical analysis for all data was performed using SPSS 27, and whether there were significances in the differences in the data sets between elephants on reunifications and unifications was calculated. Utilising the Kolmogorov–Smirnov test, it was determined whether the data distribution was normal, followed by intercorrelation calculations (Spearman’s *ρ*) of the subscales [56,57]. As the data of both the behaviour analysis and the distance analysis showed no even distribution of significance (*p* ≤ 0.05) [58,59], the data sets were not normal in distribution and the Mann–Whitney *U* Test was used to determine the significant differences (*p* ≤ 0.05) [55,56,57,58] between (re)unifications.

For the analysis of the signs shown in the *Greeting Ceremony,* a Chi-Square Test was performed and the Fisher’s Exact Test was used to detect the significance, as the data sets partially had less than size items and the effect size was calculated utilising the Monte Carlo Simulation (*x^2^*) [60,61].

The distribution for the data set of affiliative and agonistic behaviours was normal, and a t-test and the Levene’s Test for Equality of Variances was calculated to determine the significance in the differences between related and unrelated elephants during (re)unifications [51,62,63,64].

As the data for the measurement of first trunk contact during (re)unifications were distributed evenly according to the Kolmogorov–Smirnov test, an unpaired t-test and the Levene’s Test for Equality of Variances were calculated to show the significant differences between the two sample groups [51,62,63,64,65].

The distribution differed between both groups for the shown distinct behaviours during the (re)unifications, (Kolmogorov–Smirnov *p <* 0.05); therefore, the Mann–Whitney U Test was used to determine if there were significant differences in greeting behaviour [61,62,63,64].

The distribution between both groups for the data set *distance to fence* differed (Kolmogorov–Smirnov *p <* 0.05); thus, the Mann–Whitney U Test was calculated again, to determine if there were differences in the distance that the elephants kept from the fence between related and unrelated elephants [55,56,57,58].

The effect size was calculated with Pearson’s correlation coefficient: $r=\frac{z}{\surd n}$ [57,59]. For all tests, the significance level was set at *p* ≤ 0.05 [65].

## 3. Results

### 3.1. Signs of Greeting Ceremony and General Behaviour during (Re)Unifications

Based on the behavioural components of the *Greeting Ceremony*, listed in Table 1, a first analysis was performed to determine if elephants expressed the typical signs of the *Greeting Ceremony* during (re)unifications. Table 4 summarises the results and shows that all elephants that were reunited showed every behavioural item of the *Greeting Ceremony.* The behavioural items of the *Greeting Ceremony* that were also shown by all of the elephants on unifications were *raising head* and that with minor exceptions of one to two elephants were *touching trunk*, *lifting tail*, and *glandular secretion*. Only one elephant on unification emitted acoustic signals and the behavioural items *running towards elephant*, *clicking tusks, entwine trunks together*, *opening mouth*, *touching head*, *spinning around*, and *defecating/urinating* were not shown by elephants on unifications.

The statistical analysis of the data shows a significant difference for the behavioural items running towards each other, clicking of tusks, entwining trunks together, opening mouth, touching head, spinning round, acoustic signals, and defecating and urinating. There was no significant difference for the items touching trunk, folding, lifting, spreading, flapping ears, raising head (is a constant), lifting tail, and glandular secretion.

**Figure 1:**
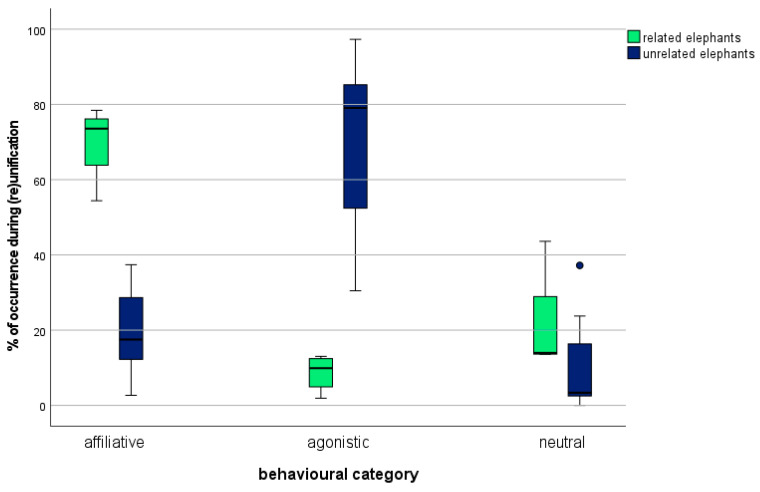
Percentage of affiliative and agonistic behaviours of the total behaviour shown by related and unrelated elephants during (re)unifications.

The results for affiliative and agonistic behaviours based on the ethogram in Table 2 show that elephants on reunification showed ~79.52% of the affiliative and ~19.65% of the agonistic behaviours while ~0.82% was neutral behaviour, and that unrelated elephants showed ~12.5% of the affiliative, ~85.08% of the agonistic, and ~2.41% of the neutral behaviours during unification (Figure 1).

Levene’s Test shows no statistical significance for the category *affiliative behaviour* (0.568); therefore, equal variance is given. The t-test shows that the mean time of affiliative behaviour was more than 50% higher for related elephants (95%-CI [33.30641, 66.87859]) than for unrelated elephants. There was a statistically significant difference between the time that the two groups expressed *affiliative behaviour*: *t*(9) = 6.751, *p* = 0.001, *d* = 4.231. For the category *agonistic behaviour,* the variance is unequal. The *t*-test shows that the mean time of *agonistic behaviour* was more than 60% lower for related elephants (95%-CI [−82.62850, −37.75650]). There was a statistically significant difference of *t*(9) = −6.370, *p* = 0.001, *d* = −3.026 (Table 5).

### 3.2. Distance to Fence during (Re)Unification

**Figure 2:**
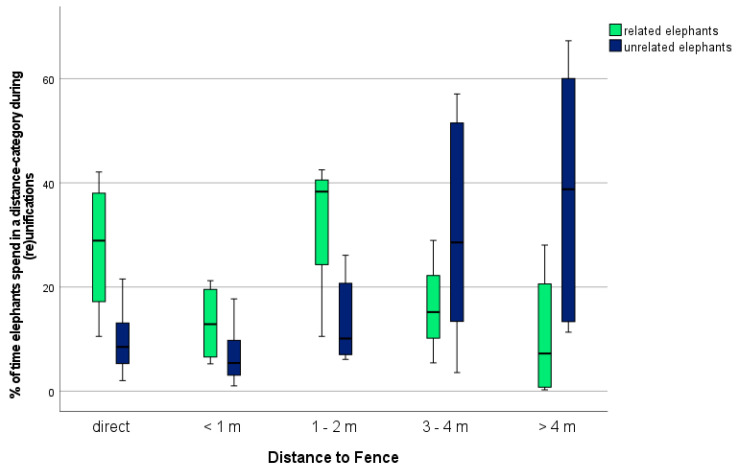
Percentage of time that related and unrelated elephants stood at a certain distance to the fence during (re)unification.

The percentage of time that the elephants spent at a certain distance to the fence at first encounter with the (un)related elephant/s is presented in Figure 2. Elephants reuniting spend ~28.31% of time in direct contact, while elephants uniting for the first time spend ~10.23% of time in direct contact. For the category <1 m, the percentages were ~23.19% (related elephants) and ~7.93% (unrelated elephants); for 1–2 m, they were ~30.12% (related) and ~15.17% (unrelated); for 3–4 m, they were ~13.05% (related) and ~33.18% (unrelated); and for >4 m, they were ~5.32% (related) and ~33.49% (unrelated).

There was a statistically significant difference in the distance to the fence in the categories *direct* and 1–2 m but not in the categories <1 m, 3–4 m, and >4 m (Table 6).

### 3.3. First Contact of Trunks

The time until first contact of trunks is shown in Table 7. Related elephants demonstrated instant contact of trunks, whilst the time until trunk contact in unrelated elephants ranged from ~100 s to more than 900 s. The elephants Umbu and Drumbo did not touch trunks during unification. Therefore, a value is not shown for this pair.

Table 8 shows the statistical differences between the two test groups for first contact of trunks. The Levene’s Test yields no statistical significance (0.165); therefore, equal variances are given. The t-test shows that the mean time until first contact of trunks was −1023.25 s (95%-CI [−3456.35, 1409.85]) lower for the related elephants than for the unrelated elephants. The difference between time until first contact of trunks for related and unrelated elephants during (re)unifications was statistically significant, *t*(10) = −2.453, *p* = 0.034.

## 4. Discussion

### 4.1. Signs of Greeting Ceremony and General Behaviour during (Re)Unifications

Free-ranging elephants live in a complex fission–fusion society, and separations and unifications are common events [28,47]. Zoo elephants, in contrast, live in stable groups, and re-unifications of related animals are very rare. We used the opportunity to monitor the exceptional situations of the reunification of two mother–daughter pairs and compared them to the unifications of six unrelated females. The results presented here are the first to describe and analyse the occurrence of behaviours displayed in both situations at first encounters in zoo elephants. We found differences in the *Greeting Ceremony* expressed for elephants united and reunited. While all elephants on reunification expressed all behavioural items described for the *Greeting Ceremony* [7,18,28,29,30,31], elephants on unifications only showed some of those behavioural items and, therefore, not a full *Greeting Ceremony* [27]. This testifies that, even in a zoo environment, the whole ceremony is only displayed if elephants know each other. This study also attests that related elephants living ex situ express the same characteristic *Greeting Ceremony*, as African elephants living in situ. This provides signs for their species-specific evolvement and preservation of species-specific behaviour. As shown in Table 3, elephants of the study were either zoo-born or transferred to zoos at an early age of just two years. This implies that they were still too young to learn all of the behaviour of the *Greeting Ceremony* in the wild and that the shown behaviour must be genetically determined in the species. The study also confirms that African elephants living in zoos recognise family members after up to 12 years of separation [7]. This provides further evidence for the long-term memory reported also for free-ranging animals [66]. The study reveals that ex situ living elephants generally showed certain greeting behaviours, even when they were unrelated, and therefore certifies the highly social behaviour in African elephants living in zoos, which is also known for the species in situ [7,67,68,69,70,71]. The study also investigated the affiliative and agonistic behaviours shown by the elephants during (re)unifications. The results clearly prove that there is a statistically significant difference for the categories *affiliative behaviour* and *agonistic behaviour*, with related elephants expressing ~50.00% more affiliative and ~60% less agonistic behaviour during reunifications than unrelated elephants. Elephants encountered familiar animals friendly and forward going (~79.52% affiliative behaviour), while elephants on unifications were hesitant and showed predominantly agonistic behaviour (~85.08%) (see Figure 1). This confirms the significance of family bonds and the general understanding of the intense social relationships of elephants [7,45,67,68,69,70,71] and their hesitation when confronted with unfamiliar individuals, which is also known from the wild [7,47]. Elephants living in situ rely on family members when raising calves, protecting the herd, and searching for food and water [1,2,3,5,47]. The results of the study indicate that behaviour that is connected to a close family bond, such as the *Greeting Ceremony*, is generically anchored in elephants and preserved in zoo-socialised elephants. It was also observed that elephants on reunifications spend more time on the *neutral* behaviour *eating/drinking* than elephants on unifications. It can be assumed that elephants on reunifications were relaxed enough to spend time eating and drinking, as the situation did not cause them an exceedingly high amount of stress [32,33,34,35,36,37], whereas elephants being united with unfamiliar elephants did not calm down enough to eat and drink, a behaviour they display typically most of the time [32,33,34,35,36,37].

### 4.2. Distance to Fence during (Re)Unification

The analysis of the distance that the elephants kept from the fence (and therefore to the closest point of contact they could reach during (re)unification) shows that elephants being reunited lingered closer to the fence than elephants that were united. Related elephants spent most of the time during reunification at a distance under two meters from the fence, while unrelated elephants stood most of the time at a distance of three meters or more, maintaining a wider distance (see Figure 2). This shows that elephants on unifications were reluctant to approach during the unifications and did not want to get close to the unfamiliar elephant. Unknown individuals can always be a threat and elephants avoid living with individuals they are not related to [47]. Their reluctance to meet unknown elephants must therefore be considered species-specific. Equally, approaching familiar and related elephants on an encounter and especially during the *Greeting Ceremony* is species-specific for African elephants [7,18,27,28,29,30,31]. These data give further evidence for species-specific behaviour present in ex situ living African elephants and the preservation of strong family bonds. Even after several years of separation, they seek close contact with their relatives.

### 4.3. First Contact of Trunks

The results of the time until first contact of trunks during (re)unifications also show a major difference between related and unrelated elephants (see Table 7). The time until first contact of trunks for related elephants is only 3 s on average; for unrelated elephants, in contrast, it is 102,625 s, being on average 342 times higher. Of the four pairs that were observed during unification, one group did not touch trunks at all during the entire first encounter. However, the range for the time until first contact of trunks during unifications is wide in unrelated elephants. Some elephants seemed to be less hesitant to touch the unfamiliar elephants than others (Saly and Drumbo, 107 s; Zimba and Lilak, 336 s). An individual distinctive disposition can be assumed, which might originate from some elephants being more curious than others, having a different social status, being of different age (and therefore less or more experienced), or having made certain previous experiences. Generally, unrelated elephants are described to be reluctant to touch the unfamiliar elephant on first encounter, while related elephants immediately seek contact with the familiar individual [1,7,47]. This observation additionally attests to the strong bonds between mother–daughter groups, which this study also found in African elephants in zoos even after a long period of separation from each other. It also confirms that related elephants on reunifications immediately approach, reach out for, and seek tactile contact with the other animal. As the olfactory and auditory senses in elephants are highly developed [9,18], these results indicate that the individuals recognised the other animal before the moment of first direct contact and wanted to engage in tactile contact with the other individual as soon as possible. Unrelated elephants, on the other hand, are aware that they are not familiar with the other individual and therefore hesitate to engage in tactile contact.

## 5. Conclusions

Even though the number of animals in the present study is small, the data presented here give further evidence of the strong bonds between mother–daughter groups. They also testify that elephants recognise each other after long-term separation by showing a full *Greeting Ceremony*, even after living apart for up to 12 years and therefore feature a species-specific behaviour even under zoo conditions, comparable with that shown in the wild. This provides evidence of recognition of their kin for the exceptional memory of this species. Keeping mothers and daughters together to build up matrilines can be considered as an important goal in the care of elephants living in European zoos [43,44,45,46,47,48,49,50,72,73].

The strong reactions expressed by mother and daughter elephants during reunifications and the empirical data of this study, demonstrating their urge to seek contact with the related animal, testify that zoo elephants, whether wild-caught or zoo-born, still belong to those species-specific mother–daughter groups. This verifies the hypothesis that elephant cows and their female offspring are better held together and that separations should be avoided in the future, where possible, to facilitate better living conditions for the animals.

Even though unifications of unrelated female elephants are a part of the European breeding programme for African elephants, elephant transfers are not frequent events and behavioural data were missing so far. Additionally, chances to observe reunifications of family members are extremely rare. Therefore, caution must be taken when interpreting ethological data, as sample size and statistical power are limited in this study [74,75]. Our preliminary findings support the need for further research.

## Figures and Tables

**Table 1:** Behaviour expressed during a *Greeting Ceremony*.

Item	Behaviour
Running towards elephant	Elephants run towards the elephant they intend to greet.
Clicking tusks and entwining trunks together	Elephants click tusks and entwine their trunks by winding them around each other.
Touching trunk	Elephants touch the trunk of the other elephant with their trunk.
Folding, lifting, spreading, and flapping ears	Elephant’s ears are in motion by folding them back, lifting them, enfolding them, and flapping them rapidly.
Raising head	Elephants raise their heads as high as possible above their shoulders.
Opening mouth	Elephants open their mouth widely.
Touching head	Elephants touch the head of the other elephant at eyes, mouth, and temporal glands with their trunk.
Spinning round	Elephants rapidly turn around repeatedly, also changing direction.
Lifting tail	Elephants lift their tail to stick it out.
Acoustic signals	Elephants emit loud vocalisation as oral rumbles, roars, and trumpets.
Defecating and urinating	Elephants drop faeces and micturate.
Glandular secretion	Elephants exude fluid from the temporal glands.

**Table 2:** Affiliative, agonistic, and neutral behaviours of greetings.

**Affiliative**	
Running towards fence/animal	Elephants run towards the elephant they intend to greet or the fence separating them from the elephant.
Pushing against the fence	Elephants press their head or body against the fence to touch the other elephant/
Touching trunks	Elephants touch the trunk of the other elephant with their trunk.
Affiliative agitation	Elephants raise their head, shake the tail, click their tusks, and flap with their ears.
Acoustic signals	Elephants emit rumbles (low-frequent calls).
Defecating/urinating	Elephants drop faeces and micturate.
**Agonistic**	
Agonistic agitation	Elephants shake the head, stick out the tail, role in their trunk, and fold their ears close to their head.
Acoustic signal	Elephants emit roars (high-frequent calls).
Pacing backwards	Elephants quickly diverge from fence/other elephants.
Showing servility	Elephants bow their head, lower their shoulders, furl the trunk, and jam their tail between their hind legs.
Showing dominance	Elephants stand tall, with raised heads and spread ears; they lift their trunk over their heads; they place the trunk on the other elephants’ head; and they run towards other elephants with sudden speed.
**Neutral**	
Eating/ drinking	Elephants eat and/or drink.

**Table 3:** List of elephants.

Elephant	Sex	Origin	Date of Birth	Date of Transfer from Wild to the Zoo	Transferred from to	Related to (Only Elephants Included in the Study Are Listed)	(Re)united with
Panya	F	Zoo-born	22 August 2007	-	Bergzoo Halle to Serengeti Park Hodenhagen	Daughter of Bibi	Bibi
Bibi	F	Wild-born	1985	1987	-	Mother of Panya	Panya
Pori	F	Wild-born	1981	1983	Tierpark Berlin to Bergzoo Halle	Mother of Tana	Tana
Tana	F	Zoo-born	4 May 2001	-	-	Daughter of Pori	Pori
Lilak	F	Wild-born	1971	1973	Tierpark Berlin to Opel-Zoo Kronberg	-	Zimba
Kariba	F	Zoo-born	17 March 2006	-	Tierpark Berlin to Opel-Zoo Kronberg	-	Zimba
Zimba	F	Wild-born	1982	1984	-	-	Kariba and Lilak
Drumbo	F	Wild-born	1990	1992	Zoo Vienna Schönbrunn to Safaripark Dvur	-	Saly, Umbu
Saly	F	Wild-born	1982	1984	-	-	Drumbo
Umbu	F	Wild-born	1981	1983	-	-	Drumbo

**Table 4:** Differences in expressed behaviour during (re)unifications.

Unrelated							Related				Exact Sig. (2-Sided) (Fisher’s Exact Test)	Effect Size (Monte Carlo Simulation)
Behaviour	Saly	Umbu	Drumbo	Zimba	Kariba	Lilak	Bibi	Panya	Tana	Pori	*p*	*X^2^*
Running towards elephant	-	-	-	-	-	-	+	+	+	+	0.003	11.00
Clicking tusks, entwine trunks together	-	-	-	-	-	-	+	+	+	+	0.003	11.00
Touching trunk	-	+	+	+	+	+	+	+	+	+	1.0	0.629
Folding, lifting, spreading, flapping ears	+	-	+	+	+	+	+	+	+	+	1.0	0.629
Raising head	+	+	+	+	+	+	+	+	+	+	-	-
Opening mouth	-	-	-	-	-	-	+	+	+	+	0.003	11.00
Touching head	-	-	-	-	-	-	+	+	+	+	0.003	11.00
Spinning round	-	-	-	-	-	-	+	+	+	+	0.003	11.00
Lifting tail	+	-	+	+	+	+	+	+	+	+	1.0	0.629
Acoustic signals	-	-	-	-	+	-	+	+	+	+	0.015	7.543
Defecating and urinating	-	-	-	-	-	-	+	+	+	+	0.003	11.00
Glandular secretion	-	-	+	+	+	+	+	+	+	+	0.491	1.397

**Table 5:** Significances for affiliative and agonistic behaviours for related and unrelated elephants on behaviour during (re)unifications.

		Levene’s Test for Equality of Variances	*t*-Test for Equality of Means							Effect Size
		Sig.	t	df	Sig. (2-Tailed) *p*	Mean Difference	Std. Error Difference	95% Confidence Interval of the Difference		*d*
								Lower	Upper	
affiliative	Equal variances assumed	0.568	6.751	9	0.000	50.09250	7.42039	33.30641	66.87859	4.231
	Equal variances not assumed		7.066	7.271	0.000	50.09250	7.08876	33.45589	66.72911	
agonistic	Equal variances assumed	0.010	−4.827	9	0.001	−60.19250	12.46980	−88.40116	−31.98384	−3.026
	Equal variances not assumed		−6.370	6.862	0.000	−60.19250	9.44953	−82.62850	−37.75650	

**Table 6:** Significances for distance to the fence between related and unrelated elephants during (re)unification.

^a^					
	Direct	<1 m	1–2 m	3–4 m	>4 m
Mann–Whitney U	3.000	6.000	3.000	10.000	5.000
Z	−2.079	−1.512	−2.079	−0.756	−1.701
Asymp. Sig. (2-tailed) *p*	0.038	0.131	0.038	0.450	0.089
Pearson’s correlation coefficient *r*	−0.627	−0.456	−0.627	−0.228	−0.513

^a^. Group variable: related, 1; unrelated, 2.

**Table 7:** Seconds until first contact of trunks during (re)unifications for the different pairs that were (re)united.

Setting	Elephant Pair	Time until Contact (s)	Average
Unification	Saly and Drumbo	107	450
	Umbu and Drumbo	not displayed	
	Zimba and Lilak	936	
	Zimba and Kariba	362	
Reunification	Bibi and Panya	2	3
	Pori and Tana	4	

**Table 8:** Significances for related and unrelated elephants on first contact of trunks during unification.

		Levene’s Test for Equality of Variances	*t*-Test for Equality of Means						
		Sig.	t	df	Sig. (2-tailed) *p*	Mean Difference	Std. Error Difference	95% Confidence Interval of the Difference	
								Lower	Upper
First Contact of Trunks	Equal variances assumed	0.002	−2.453	10	0.034	−723.250	294.809	−1380.126	−66.374

## Abbreviations

CI	Confidence interval
d	Effect size after Cohen
df	Degree of freedom
M	Mean
m	Meter
N/n	Sample size
p	Significance
r	Effect size after Pearson
SD	Standard Deviation
sec	Seconds
t	t-Statistic
U	Mann-Whitney U-Statistic
X2	Effect size after Monte Carlo
Z	Z-Statistic
%	Percent
